# (2-Hydroxypropyl)-β-Cyclodextrin Is a New Angiogenic Molecule for Therapeutic Angiogenesis

**DOI:** 10.1371/journal.pone.0125323

**Published:** 2015-05-05

**Authors:** Xun Qi, Yonghui Yuan, Ke Xu, Hongshan Zhong, Zhen Zhang, Huan Zhai, Gefei Guan, Guibo Yu

**Affiliations:** 1 Department of Radiology, The First Affiliated Hospital of China Medical University, Shenyang, China; 2 Key Laboratory of Diagnostic Imaging and Interventional Radiology of Liaoning Province, The First Affiliated Hospital of China Medical University, Shenyang, China; 3 Hospital infection management office, Liaoning Cancer Hospital & Institute, Shenyang, China; 4 Department of ultrasound, The First Affiliated Hospital of China Medical University, Shenyang, China; 5 Department of neurosurgery, The First Affiliated Hospital of China Medical University, Shenyang, China; Faculty of Biochemistry, Biophysics and Biotechnology, Jagiellonian University, POLAND

## Abstract

**Background:**

Peripheral artery disease (PAD), which is caused by atherosclerosis, results in progressive narrowing and occlusion of the peripheral arteries and inhibits blood flow to the lower extremities. Therapeutic angiogenesis is a promising strategy for treating ischemia caused by PAD. Nitric oxide (NO) has been shown to be a key mediator of angiogenesis. It has been demonstrated that β-cyclodextrincan stimulate vessel growth in rabbit corneas. In this study, we assessed the mechanism of action and therapeutic potential of a new angiogenic molecule, (2-hydroxypropyl)-β-cyclodextrin (2HP-β-CD).

**Methods and Results:**

2HP-β-CD significantly increased vascular endothelial growth factor A (VEGF-A) and platelet-derived growth factor BB (PDGF-BB) peptides in human umbilical vein endothelial cells (HUVECs) and also increased basic fibroblast growth factor (bFGF) peptide in human aortic smooth muscle cells (HASMCs). 2HP-β-CD stimulated both proliferation and migration of HUVECs in an endothelial nitric oxide synthase (eNOS)/NO-dependent manner, whereas NO was found to be involved in proliferation, but not migration, of HASMCs. In a unilateral hindlimb ischemia model in mice, 2HP-β-CD injections not only promoted blood flow recovery and increased microvessel densities in ischemic muscle, but also promoted coverage of the vessels with smooth muscle cells, thus stabilizing the vessels. Administration of 2HP-β-CD increased the expression of several angiogenic factors, including VEGF-A, PDGF-BB and transforming growth factor beta-1 (TGF-β1) in ischemic muscle. Injections of 2HP-β-CD also stimulated protein kinase B and extracellular regulated protein kinases (ERK), leading to an increase in phosphorylation of eNOS in ischemic muscle. Treatment with the NOS inhibitor, Nω-nitro-L-arginine methyl ester (L-NAME), showed that stimulation of blood flow induced by 2HP-β-CD was partially dependent on NO.

**Conclusions:**

Therapeutic angiogenesis by 2HP-β-CD may be beneficial to patients with PAD.

## Introduction

Peripheral artery disease (PAD), which is caused by atherosclerosis, results in progressive narrowing and occlusion of the peripheral arteries and inhibits blood flow to the lower extremities [1]. PAD is a highly prevalent syndrome that affects 8–12 million individuals in the United States and is associated with significant morbidity and mortality [2,3]. Global prevalence of PAD is 3%–10%, increasing to 15%–20% in individuals over the age of 70 [4]. Critical limb ischemia (CLI) is a severe form of PAD characterized by ischemic rest pain with ischemic ulcers and/or gangrene [5]. The primary therapeutic goals in treating CLI are to reduce the risk of adverse cardiovascular events, relieve ischemic pain, heal ulcers, prevent major amputation, and improve quality of life and survival. These goals may be achieved by medical therapy, endovascular intervention, open surgery, or amputation and require a multidisciplinary approach including pain management, wound care, reduction of risk factors, and treatment of co-morbidities [6]. Approximately 20%–30% of patients with CLI are not, however, considered to be suitable candidates for vascular or endovascular procedures and amputation is often the only option [7]. This corresponds to about 100,000 major leg amputations in the European Union, and to 120,000 in the United States [8]. These no-option patients are potential candidates for novel angiogenic therapies.

Many pharmacological approaches, based mainly on the knowledge gained from studying therapeutic angiogenesis, have been used to stimulate the growth of collateral arteries [9]. Approaches for therapeutic angiogenesis can be divided into three categories: (1) protein-based, (2) gene-based, and (3) cell-based therapies [10,11]. All of these approaches have been used to achieve therapeutic angiogenesis in cardiac and skeletal muscle [12,13]. But each approach has disadvantages. The disadvantages of using recombinant proteins include the considerable costs involved in producing significant quantities of pyrogen-free material as well as the requirement for repeated or prolonged administration of protein because of its short half-life [14]. Disadvantages of gene therapy include relatively low transfection rates (especially in skeletal muscle), potential for uptake of vector and gene expression at distant sites with unwanted effects in non-target tissues and the inability to withdraw treatment quickly [15,16]. Most recently, cell-based therapies have emerged as exciting avenues for therapeutic angiogenesis. Endothelial progenitor cells (EPCs) have been found to participate in postnatal neovascularization after mobilization from bone marrow [17]. Although treatment of vascular occlusive disease with peripheral blood-derived cultured EPCs might be a potential therapeutic option, estimates based on animal experiments suggest that 12L of autologous blood may be necessary to harvest sufficient EPCs to induce angiogenesis in patients if the EPCs are delivered *via* an intravenous infusion [18]. Because such an approach is impractical in a clinical setting, approaches to render EPC therapy more feasible are currently being developed. It is therefore crucial that new angiogenic molecules are identified and tested for treating PAD.

Recently, NO produced by eNOS has been shown to play a crucial role in the regulation of vascular tone, vascular remodeling, and angiogenesis [19,20].

Cyclodextrins are cyclic oligosaccharides consisting of six, seven, or eight glucopyranose units, usually referred to as α-, β-, or γ-cyclodextrins, respectively [21]. Glycosaminoglycans play a role in vascular endothelial cell function as they are able to modulate the activities of angiogenic growth factors by facilitating interactions with their receptors and promoting receptor activation [22]. It has been demonstrated that β-cyclodextrin, administered alone, stimulated vessel growth to 164% of the growth in untreated controls and vessel density to 303% above the control level in rabbit corneas [23]. In contrast, it has also been reported that sulfated β-cyclodextrin acted as an angiogenesis inhibitor when administered at high concentrations [24]. The role of NO in cell migration and proliferation induced by 2HP-β-CD and the effect of 2HP-β-CD in a mouse hindlimb ischemia model are, however, unknown.

In the present study, we investigated the functions and mechanisms of action of 2HP-β-CD in angiogenesis both in vitro and in vivo. We showed that 2HP-β-CD significantly increased VEGF-A and PDGF-BB peptides in HUVECs and bFGF peptide in HASMCs. 2HP-β-CD stimulated proliferation and migration of HUVECs in an eNOS/NO-dependent manner, whereas NO was found to be involved in proliferation, but not migration, of HASMCs. Local injections of 2HP-β-CD into ischemic limb muscle promoted recovery of blood flow, with stimulation of microvessel formation and vascular maturation. These therapeutic effects of 2HP-β-CD were dependent on Akt/ERK-eNOS. Treatment with NOS inhibitor, L-NAME, showed that stimulation of blood flow induced by 2HP-β-CD was partially dependent on NO. These observations provide the basis for proposing 2HP-β-CD as a novel treatment for post-ischemic angiogenesis.

## Materials and Methods

### Materials

2HP-β-CD, pentobarbital, L-NAME and bovine serum albumin (BSA) were purchased from Sigma-Aldrich (St. Louis, USA). Crystal violet was purchased from Biotime (Jiangsu, P.R.China).

### Animals

Male C57BL/6 mice, aged 8–12 weeks, were obtained from the Animal Facility at the China Medical University. Mice were housed in a conventional temperature-controlled facility (24°C) under a 12:12 h light–dark cycle with free access to regular chow and water. Animal care and experimental procedures were approved by the Animal Care and Use Committee of the China Medical University.

### Cell Culture

HUVECs were obtained from KeyGEN BioTECH (Nanjing, P.R. China). The cells were cultured in RPMI 1640 (Thermo Scientific, Rockford, USA) containing 10% fetal bovine serum (FBS) (Thermo Scientific), 100 IU/ml penicillinand 100 mg/ml streptomycin (KeyGEN BioTECH).

HASMCs were obtained from Chinese academy of sciences, Shanghai Institutes for Cell Resource Center (Shanghai, P.R. China). The HASMCs were then grown in Dulbecco’s modified Eagle’s medium (DMEM) (Thermo Scientific) supplemented with 10% FBS (Thermo Scientific), 100 U/ml of penicillin, and 100 mg/ml of streptomycin (KeyGEN BioTECH).

### Transwell Migration Assay

Transwell migration of HUVECs and HASMCs was determined using a 24-well transwell chamber with a pore size of 8 μm (Millipore, Massachusetts, USA). Cells (1x10^5^) in 200 μl of serum-free RPMI 1640 or DMEM containing 0.25% fatty acid-free BSA were loaded into the upper wells; the lower wells were filled with the same medium with or without 10^-8^M 2HP-β-CD and/or 1mM L-NAME. The cells were allowed to migrate across the porous filter for 5 h at 37°C in a tissue culture incubator. After staining with 1% crystal violet and scraping the upper membrane surface with a cotton tip, the number of cells that migrated to the lower side of the filter was counted under a microscope in ten randomly selected high power fields. This experiment was performed in triplicate.

### Cell Proliferation Test

A cell proliferation assay was performed using Cell Counting Kit-8 (CCK-8) solution (Dojindo, Gaithersburg, MD) according to the manufacturer’s protocol. Briefly, HUVECs were seeded at a concentration of 1x10^3^ cells/100 μl/well and HASMCs were seeded at a concentration of 1.5x10^3^ cells/100 μl/well in 96-well culture plates. The cells were incubated with or without 10^-8^M 2HP-β-CD and/or 1 mM L-NAME for 72 h. The wells were treated with 10 μl/well of CCK-8 solution during the last 4 h of culture. Optical density (OD) of the wells was measured at 450 nm using a microplate reader (Thermo Scientific). This experiment was performed in triplicate.

### Unilateral Hindlimb Ischemia Model in Mice

Following intraperitoneal injection of pentobarbital (60 mg/kg), mice were subjected to surgical procedures to achieve unilateral hindlimb ischemia according to the method described previously [25]. In brief, following a skin incision at the left paracenter of the lower abdomen, the femoral artery, which originates from the external iliac artery and terminates to bifurcate into the saphenous and the popliteal arteries, was exposed. The femoral artery was ligated with 8–0 silk, and the whole length of the femoral artery was excised and the skin incision sutured.

### Blood Flow Analysis

Blood flow in the ischemic hindlimb was measured using a laser Doppler blood flow (LDBF) imager (Moor Instruments, Devon, UK) before and after the operation. Recovery of perfusion in the ischemic hindlimb of each mouse was estimated by the percentage of limb blood flow, calculated as the average perfusion of the left hindlimb compared to that of the normal right hindlimb.

### Drug Administration

2HP-β-CD (10^–7^ M, 10^–8^ M and 10^–9^ M) was suspended in Dulbecco’s phosphate-buffered saline (PBS). The suspensions of 2HP-β-CD (10 μl for each site) or PBS alone (10 μl for each site) were injected intramuscularly into four sites in the medial portion of the thigh muscle and two sites in the calf muscle of ischemic limbs daily for 28 days.

L-NAME (0.5 mg/ml) was dissolved in drinking water containing 1% glucose and administered to mice for 4 weeks before and after the operation; control animals received drinking water containing 1% glucose. This dose of L-NAME was chosen because it was previously shown to induce hypertension through inhibition of NO production [26]. All animals were fed a regular chow diet. Systolic and diastolic blood pressure and heart rate were measured in conscious mice by the tail–cuff method using an indirect blood pressure meter (BP-98A, Softron Co, Tokyo, Japan) according to manufacturer's protocol.

### Immunohistochemistry and Immunofluorescence

Mice were perfused with PBS containing 4% paraformaldehyde through a cannula inserted into the left ventricle. The calf muscle was excised, embedded in O.C.T. compound (Sakura Fine Chemical, Tokyo, Japan), frozen on dry ice, and sectioned (8 μm). For immunofluorescence measurements, acetone-fixed frozen sections were incubated with rat monoclonal anti-CD31/PECAM-1 antibody (1:100, clone MEC13.3, BD Biosciences, San Jose, CA) and mouse monoclonal anti-αsmooth muscle actin (αSMA) (1:100, clone 1A4, Sigma). Bound antibodies were detected using TRITC-conjugated goat anti-rat IgG (Proteintech, Chicago, USA) and FITC-conjugated goat anti-mouse IgG (Proteintech) secondary antibodies. For immunohistochemistry, tissue sections probed with primary antibodies were incubated with StreptAvidin-Biotin Complex (SABC) kit, (Boster, Wuhan, P.R. China), followed by visualization with 3,3’–diaminobenzidine tetrahydrochloride (Maxim, Fuzhou, P.R. China).

CD31-positive capillary densities were counted in ten randomly chosen high power fields per mouse and expressed as the number of capillaries per mm^2^. Quantification of the extent of αSMA-positive microvasculatures was carried out using an Image J (NIH) system.

### Isolation of mRNA and Quantitative Real-time Polymerase Chain Reaction

Cells were incubated with or without 10^-8^M 2HP-β-CD for 8 h. Total RNA from cells and calf muscle was isolated using RNAiso Plus (TAKARA Biotechnology, Dalian, P.R. China). The concentration of RNA was determined by spectrophotometry at 260 nm. Total RNA (1 μg) was reverse-transcribed using a PrimeScript RT reagent Kit (Perfect Real Time) (Takara) in a total volume of 20 μl. Quantitative real-time PCR was performed on cDNA samples using a LightCycler480 II instrument (Roche, Basel, Switzerland) with the SYBR Green Premix Ex Taq II (Takara) in a total volume of 20 μl. The primer sequences for cells were as follows: Vegfa-F, 5′- GACTCGCCCTCATCCTCTTC-3′; Vegfa-R, 5′- TCAACCACTCACACACACACAAC-3′; Fgf2-F, 5′- GTGTGCTAACCGTTACCTGGCTATG-3′; Fgf2-R, 5′- CCAGTTCGTTTCAGTGCCACA-3′; Pdgfb-F, 5′- GAAATTCAAGCACACGCATGAC-3′; Pdgfb-R, 5′- TCCGAATCAGGCATCGAGAC-3′; Tgfb1-F, 5′- CGCATCCTAGACCCTTTCTCCTC-3′; Tgfb1-R, 5′- GGTGTCTCAGTATCCCACGGAAAT-3′; Angpt1-F, 5′- TCAACATCTGGAACATGTGATGGA-3′; Angpt1-R, 5′- CATGGTAGCCGTGTGGTTCTG-3′; GAPDH-F, 5′-GATCTCGCTCCTGGAAGATG-3′; and GAPDH-R, 5′-CAATGGCCCCTTCATTGACC-3′. The primer sequences for tissues were as follows: Vegfa-F, 5′-AGGCAGACTATTCAGCGGATCC-3′; Vegfa-R, 5′-CTCAAACCGTTGGCACGA-3′; Fgf2-F, 5′-GGCTGCTGGCTTCTAAGTG-3′; Fgf2-R, 5′-CTGTCCAGGTCCCGTTTT-3′; Pdgfb-F, 5′-CACCAACGCCAACTTCCT-3′; Pdgfb-R, 5′-GCTTCTTTCGCACAATCTCA-3′; Tgfb1-F, 5′-AACTAAGGCTCGCCAGTCC-3′; Tgfb1-R, 5′-GCGGTCCACCATTAGCAC-3′; Angpt1-F, 5′-ATTCTTCGCTGCCATTCT-3′; Angpt1-R, 5′-CAGTTCCCGTCGTGTTCT-3′; GAPDH-F, 5′-CTCTGCTCCTCCCTGTTC-3′; and GAPDH-R, 5′-CAATCTCCACTTTGCCACT-3′. The expression level of glyceraldehyde-3-phosphate dehydrogenase (GAPDH) was measured and used as an internal control. The data were analyzed using 2^-ΔΔCt^ relative expression quantity as previously described [25].

### Enzyme Linked ImmunoSorbent Assay (ELISA)

The concentrations of VEGF-A, PDGF-BB and bFGF in the cell supernatants were determined using a human VEGF-A ELISA kit (Dakewe Biotech, Shenzhen, P.R. China), a human factor-BB ELISA kit (Neobioscience, Shenzhen, P.R. China) and a human bFGF ELISA kit (Neobioscience), respectively. All ELISA kits were used according to the manufacturers' instructions. VEGF-A, PDGF-BB and TGF-β1 in the tissue lysates were determined using a mouse VEGF-A Platinum ELISA kit (eBioscience, Vienna, Austria), a mouse platelet-derived growth factor-BB ELISA kit (Cusabio, Wuhan, P.R. China) and a mouse TGF-β1 Platinum ELISA kit (eBioscience), respectively. Briefly, cells were incubated with or without 10^–8^ M 2HP-β-CD for 24 h, the supernatants were collected and centrifuged at 5000 *g* for 10 min, and the supernatants were collected for further analysis. Calf muscle tissues were dissected out and homogenized in lysis buffer containing protease inhibitors. The homogenized samples were centrifuged at 5000 *g* for 10 min, and the supernatants were collected for further analysis; Total protein concentrations were subsequently measured using a bicinchoninic acid (BCA) protein assay (Thermo Scientific). VEGF-A, PDGF-BB, bFGF and TGF-β1 ELISA kits were used to establish standard curves. The results were reported in picograms per mg of proteins and compiled using Prism 6.0 GraphPad Software (GraphPad Software Inc., San Diego, CA).

### Western Blotting

Cells were incubated with or without 10^–8^ M 2HP-β-CD and/or 1 mM L-NAME for 30 min. Total proteins from cells were extracted in M-PER mammalian protein extraction reagent (Thermo Scientific) supplemented with Halt Protease and Phosphatase Inhibitor Cocktail (Thermo Scientific). Excised muscle tissues were snap-frozen in liquid nitrogen and homogenized in M-PER mammalian protein extraction reagent (Thermo Scientific) supplemented with Halt Protease and Phosphatase Inhibitor Cocktail (Thermo Scientific). After removal of debris by centrifugation (800 *g* at 4°C for 15 min), the supernatants were used for western blot analysis. Totals of 20 μg proteins (for cells) and 60 μg proteins (for tissues) were loaded and separated on 4%–12% gradient mini gels (Thermo Fisher, Waltham, MA, USA) and electrotransferred onto polyvinylidene difluoride (PVDF) membranes (Thermo Fisher). The membranes were blocked with 5% milk or 5% BSA powder (BD Bioscience, San Jose, CA), washed with Tris Buffered Saline with Tween 20 (TBST) (Sigma-Aldrich), and incubated overnight at 4°C with rabbit polyclonal anti-Ser^1177^-phosphorylated-eNOS antibody (1:1000, Cell Signaling, Danvers, MA), rabbit polyclonal anti-Ser^473^-phosphorylated-Akt antibody (1:1000, Cell Signaling), rabbit polyclonal anti-Thr^202^, Tyr^204^-phosphorylated-ERK antibody (1:1000, Cell Signaling), rabbit polyclonal anti-eNOS antibody (1:1000, Cell Signaling), rabbit polyclonal anti-Akt antibody (1:1000, Cell Signaling), and rabbit polyclonal anti-ERK antibody (1:2000, Cell Signaling). The membranes were washed with TBST and incubated with peroxidase-conjugated anti-rabbit or anti-mouse IgG (Santa Cruz Biotechnology, Santa Cruz, CA) as a secondary antibody. The membranes were washed with TBST, and developed using SuperSignal West Femto Maximum Sensitivity Substrate (Thermo Scientific). The protein signal was imaged and analyzed using Image Lab 4.1 software (BioRad, Hercules, CA).

### Statistics

All values are expressed as the mean ± S.E.M. (standard error of mean). The data were analyzed using a one-way ANOVA followed by a Bonfferoni post test (Prism 6.0 GraphPad Software). Statistical significance between two groups was analyzed by Student's *t* test. Values of *p* < 0.05 were considered to be statistically significant.

## Results

### Administration of 2HP-β-CD Upregulates Angiogenesis-related Gene and Peptide Expression in HUVECs and HASMCs

**Fig 1:**
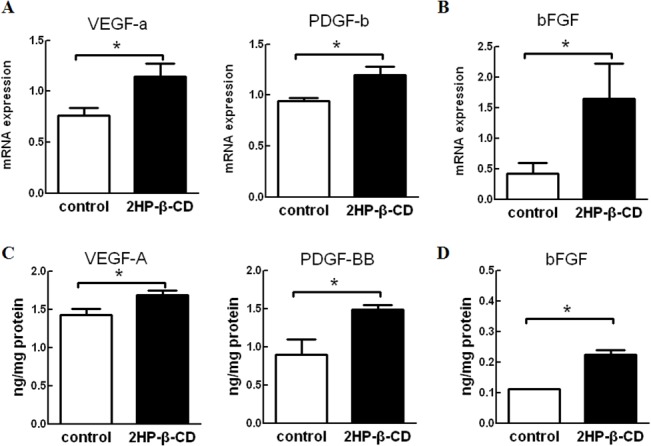
Angiogenesis-related mRNA and peptide expression in HUVECs and HASMCs. Expression levels of mRNA for angiogenic factors in HUVECs (A) and HASMCs (B) when treated with 2HP-β-CD (filled) and PBS (open) were determined by real-time PCR. GAPDH was used as an endogenous control. Data represent the mean ± S.E.M. (*n* = 4 per group). * *p* < 0.05. Angiogenesis-related peptide expression in the supernatants of HUVECs (C) and HASMCs (D) when treated with 2HP-β-CD (filled) and PBS (open) were determined by ELISA. Data are reported in nanograms per mg of proteins and represent the mean ± S.E.M. (*n* = 3 experiments). * *p* < 0.05.

Our preliminary experiments showed that 10^–8^ M 2HP-β-CD significantly increased cell migration and proliferation in both HUVECs and HASMCs. To study the mechanisms of 2HP-β-CD-induced cell migration and proliferation, the effects of 2HP-β-CD on expression level of mRNA for various angiogenic factors were examined by real time-PCR analysis. Among the angiogenic factors examined, expression of both VEGF-A and PDGF-B mRNA were increased in HUVECs (Fig 1A), and expression of bFGF mRNA was increased in HASMCs (Fig 1B) when treated with 10^–8^ M 2HP-β-CD. We next sought to determine whether VEGF-A, PDGF-BB and bFGF peptide levels were also increased following administration of 10^–8^ M 2HP-β-CD. Significant increases in VEGF-A and PDGF-BB peptides (Fig 1C) in HUVECs and bFGF peptide in HASMCs were observed (Fig 1D).

### eNOS Phosphorylation is Induced by 2HP-β-CD

**Fig 2:**
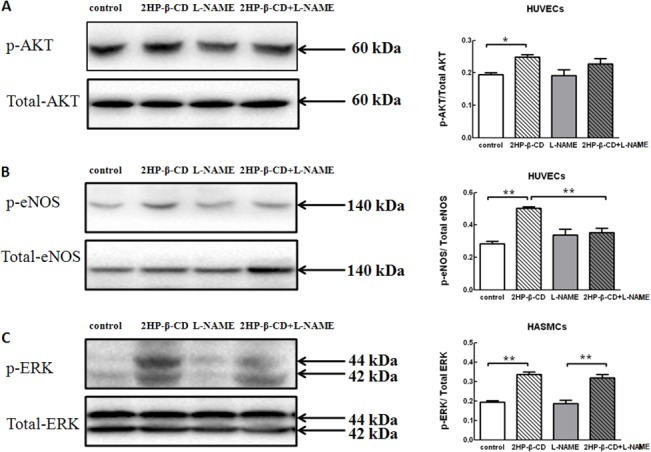
Phosphorylation of Akt, ERK and eNOS in HUVECs and HASMCs following treatment with 2HP-β-CD or PBS. HUVECs and HASMCs were pretreated with/without L-NAME followed by incubation with/without 2HP-β-CD for 30 min. Phospho-Akt and total Akt in HUVECs (A), phospho-eNOS and total eNOS in HUVECs (B) and phospho-ERK and total ERK in HASMCs (C) were assessed by western blot. The quantitative analyses of band densities are also shown. Data represent the mean ± S.E.M. (*n* = 4 experiments). * *p* < 0.05, ** *p* < 0.01.

Previous studies have shown that cholesterol-free methyl-β-cyclodextrin stimulates eNOS [27]. Akt phosphorylates eNOS and thereby enhances activity [28]. ERK1/2 has also been shown to stimulate eNOS by phosphorylation [29]. We next characterized the 2HP-β-CD-induced phosphorylation of Akt, ERK and eNOS in HUVECs and HASMCs. 2HP-β-CD increased phosphorylation of Akt (Fig 2A) and eNOS (Fig 2B) in HUVECs, and the increase of phosphorylation of eNOS was effectively blocked by pharmacological inhibition with a NOS inhibitor, L-NAME (Fig 2B). 2HP-β-CD increased phosphorylation of ERK (Fig 2C) in HASMCs, and this effect was not blocked by treatment with L-NAME. These results indicated that 2HP-β-CD promotes HUVECs proliferation and migration in a NO-dependent manner.

### Role of NO in 2HP-β-CD-induced cell Migration and Proliferation

**Fig 3:**
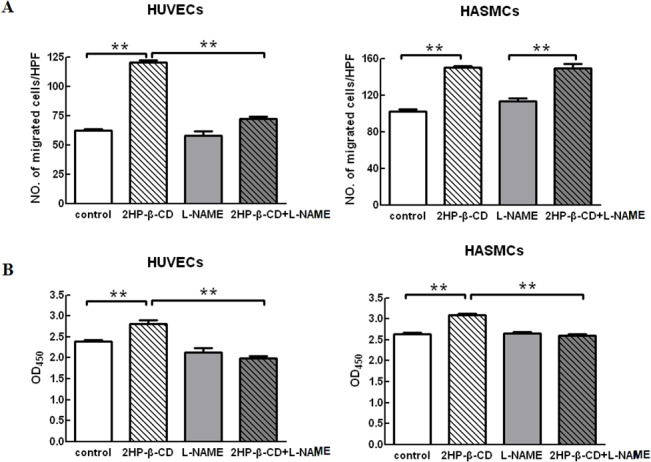
Effects of L-NAME on migration and proliferation of HUVECs and HASMCs induced by 2HP-β-CD. HUVECs and HASMCs were pretreated with/without 1 mM L-NAME followed by incubation with/without 10^-8^M 2HP-β-CD. Migration (A, 5 h) and proliferation (B, 72 h) of the cells were assessed as described in “Materials and Methods”. Data represent the mean ± S.E.M. (*n* = 3 experiments). * *p* < 0.05, ** *p* < 0.01.

To analyze the mechanism of 2HP-β-CD-induced cell migration and proliferation, we finally assessed whether NO is involved in 2HP-β-CD-stimulated migration and proliferation. As shown in Fig 3A, 2HP-β-CD stimulated cell migration in both HUVECs and HASMCs; treatment with L-NAME significantly blocked migration of HUVECs, but not that of HASMCs. 2HP-β-CD also stimulated proliferation of HUVECs and HASMCs; this effect of 2HP-β-CD was inhibited by L-NAME in both cell types (Fig 3B). These results suggest that, under our experimental conditions, eNOS activity regulates 2HP-β-CD-induced proliferation and migration of HUVECs, whereas NO is involved in proliferation, but not migration, of HASMCs.

### Local Administration of 2HP-β-CD Stimulates Blood Flow Recovery and Angiogenesis in Ischemic Hindlimbs

**Fig 4:**
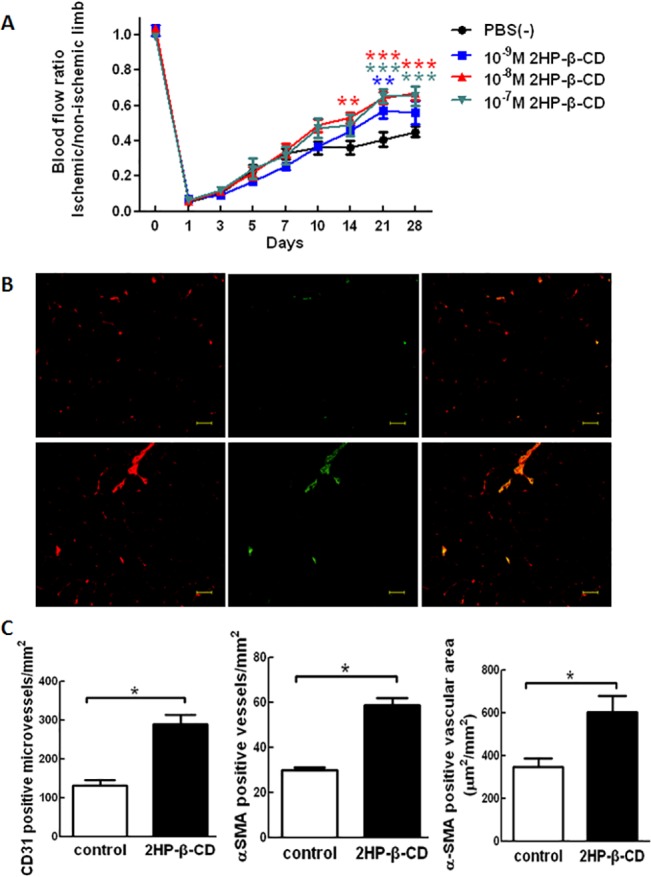
Local injection of 2HP-β-CD enhances blood flow recovery (A), and stimulates angiogenesis and arteriogenesis (B). (A) Ischemic/non-ischemic limb blood flow ratio assessed by LDBF imager in mice that received different concentrations of 2HP-β-CD for 28 days. Data represent the mean ± S.E.M. (*n* = 8 mice per group). ** *p* < 0.01 and *** *p* < 0.001 versus the PBS-treated mice. (B) 2HP-β-CD-stimulated angiogenesis and arteriogenesis in C57BL/6 mice. EC marker CD31 (red) and SMC marker αSMA (green) were detected by double immunofluorescence in the ischemic hindlimb of PBS-treated (upper panel) and 2HP-β-CD-treated (lower panel) mice. Representative immunofluorescence images of each marker and merged images are shown. (C) CD31 positive microvessels, αSMA positive vessels and αSMA positive vascular area were quantified using Image J software. Data represent the mean ± S.E.M. (*n* = 4 mice per group). * *p* < 0.05 versus the PBS-treated control mice. Scale bar = 50 μm.

The effects of different concentrations of 2HP-β-CD on blood flow in ischemic hindlimbs after surgical femoral arteriectomy in C57BL/6 mice were compared. Concentrations of 10^–8^ M and 10^–7^ M 2HP-β-CD stimulated blood flow at post-operative day 28, and at other time points. Optimal effects were observed at a concentration of 10^–8^ M. On post-operative day 1, the ratio of blood flow in the ischemic limb to that in the non-ischemic limb decreased sharply to 5% of the pre-surgery level in mice injected with 2HP-β-CD and control animals. Blood flow recovery was significantly higher in mice treated with 10^–8^ M 2HP-β-CD than in control mice from post-operative day 14 to post-operative day 28. Muscle tissues from the optimal concentration of 10^–8^ M group and control group were used for the following experiments (Fig 4A).

Double immunofluorescence using anti-CD31 (endothelial cell marker) and anti-αSMA (smooth muscle marker) antibodies showed that anti-CD31-positive staining microvessel density in calf muscle of ischemic limbs at post-operative day 28 was significantly greater in 2HP-β-CD-treated mice compared with control mice (Fig 4B). Anti-αSMA-positive blood vessels were increased in 2HP-β-CD-treated mice compared with control mice. The anti-αSMA-positive vascular cross-sectional area was also increased in 2HP-β-CD-treated mice compared with control mice, indicating that relatively larger blood vessels were formed in 2HP-β-CD-treated mice compared with control mice.

These observations indicate that administration of 2HP-β-CD promotes the formation of smooth muscle-paved larger vessels in ischemic muscle, thus inducing stabilization and growth of blood vessels.

### Administration of 2HP-β-CD Upregulates Angiogenesis-related Gene and Peptide Expression in Ischemic Hindlimbs

**Fig 5:**
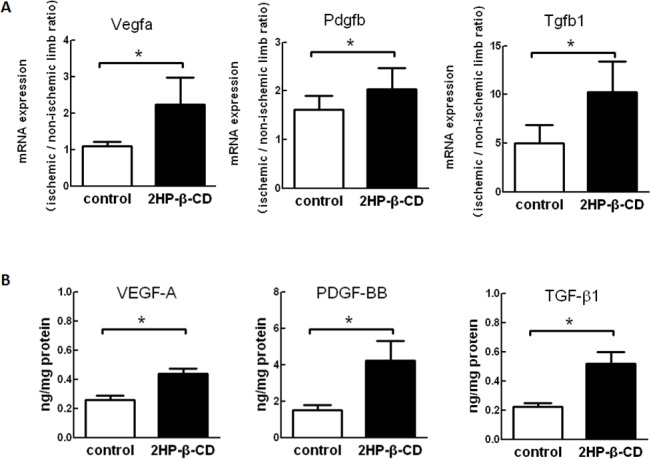
Angiogenesis-related mRNA and peptide expression in ischemic hindlimb muscle. (A) Expression levels of Mrna for angiogenic factors in calf muscle from 2HP-β-CD-treated (filled) and PBS-treated (open) mice at post-operative day 28 were determined by real-time PCR. GAPDH was used as an endogenous control. Data are expressed as the ratio of the values in ischemic over non-ischemic muscle and represent the mean ± S.E.M. (*n* = 8 mice per group). * *p* < 0.05. (B) Angiogenesis-related peptide expression in calf muscle from 2HP-β-CD-treated (filled) and PBS-treated (open) mice at post-operative day 28 were determined by ELISA. Data are reported in nanograms per mg of proteins and represent the mean ± S.E.M. (*n* = 6 mice per group). * *p* < 0.05.

To study the mechanisms of 2HP-β-CD-induced stimulation of angiogenesis and blood flow, the effects of 2HP-β-CD on expression levels of mRNA for various angiogenic factors were examined by real-time-PCR analysis. Among the angiogenic factors examined, expression levels of VEGF-A, PDGF-B and TGF-β1 mRNA were increased in ischemic limb muscle compared with non-ischemic muscle (Fig 5A). These results suggest that administration of 2HP-β-CD increases the expression of these angiogenic factors, which might contribute to stimulated angiogenesis in ischemic limbs. Having observed that VEGF-A, PDGF-B and TGF-β1 mRNA increased in ischemic limb muscle compared with non-ischemic muscle on post-operative day 28 following administration of 2HP-β-CD, we next sought to determine whether intramuscular immunoreactive VEGF-A, PDGF-BB and TGF-β1 levels also increased. Following administration of 2HP-β-CD, significant increases in VEGF-A, PDGF-BB and TGF-β1 peptides in ischemic limb muscle compared with non-ischemic muscle were seen on post-operative day 28 (Fig 5B).

These data indicate that, in addition to increased mRNA levels, VEGF-A, PDGF-BB and TGF-β1 peptide levels are also increased in ischemic limb muscle compared with non-ischemic muscle on post operative day 28 following administration of 2HP-β-CD.

### 2HP-β-CD Promotes Blood Flow Recovery in a Nitric Oxide (NO)-dependent Manner

**Fig 6:**
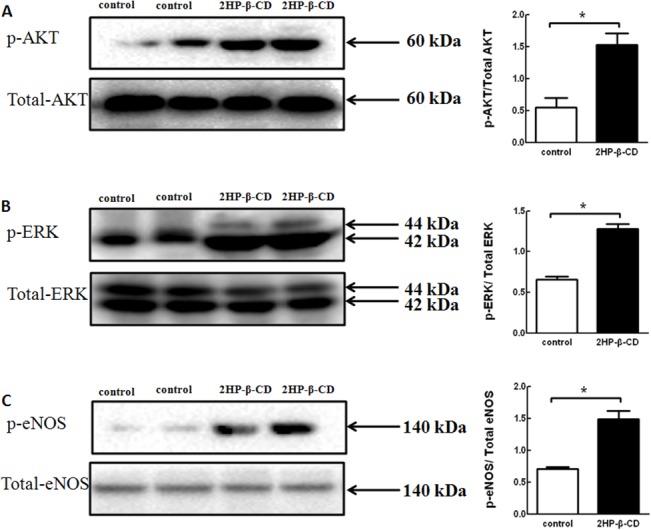
Phosphorylation of Akt, ERK and eNOS in ischemic muscle following injections of 2HP-β-CD or PBS. Muscle tissue homogenates from 2HP-β-CD-treated (filled) and PBS-treated (open) mice at post-operative day 28 were subjected to western blot using antibodies against phospho-Akt and total Akt (A), phospho-ERK and total ERK (B), and phospho-eNOS and total eNOS (C) as described in “Materials and Methods”. The quantitative analyses of band densities are also shown. Data represent the mean ± S.E.M. (*n* = 5 mice per group). * *p* < 0.05, versus the PBS-treated control mice.

The involvement of NOS in 2HP-β-CD-induced neovascularization in ischemic limbs was then examined. Stimulation of eNOS and the consequent increase in NO production contributes to post-ischemic angiogenesis and blood flow recovery [30,31]. 2HP-β-CD injections stimulated phosphorylation of both Akt and ERK, with an increase in phosphorylation of eNOS in ischemic limb muscle (Fig 6). This suggests that 2HP-β-CD probably stimulates eNOS through Akt and ERK in ischemic limb muscle *in vivo*.

**Fig 7:**
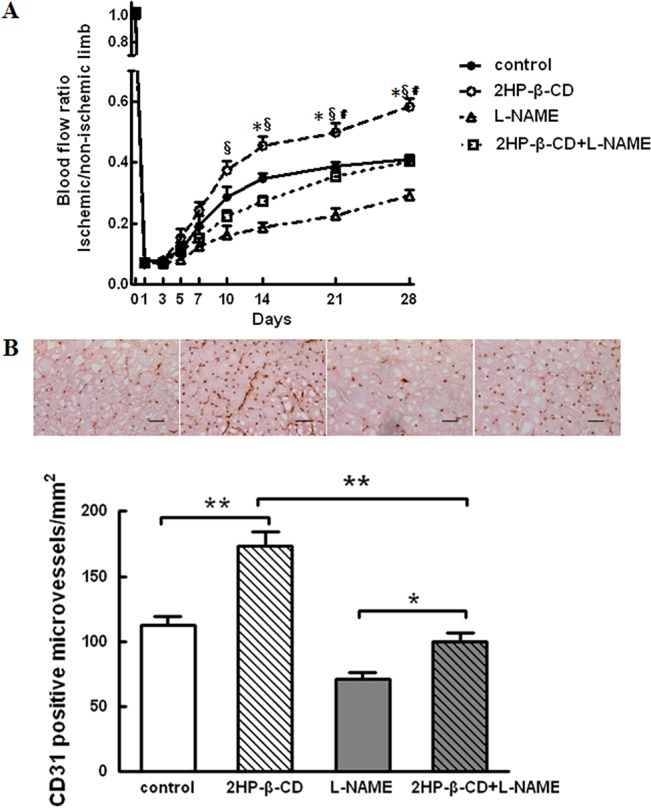
Effect of L-NAME on 2HP-β-CD induced stimulation of blood flow recovery in C57BL/6 mice. A, Ischemic/non-ischemic limb blood flow ratio assessed by LDBF imager in mice in 4 experimental groups; PBS-treated mice with or without L-NAME, 2HP-β-CD-treated mice with or without L-NAME. Data represent the mean ± S.E.M. (n = 10 mice per group). * *p <* 0.05, 2HP-β-CD-treated mice versus PBS-treated mice. *§ p <* 0.05, 2HP-β-CD-treated mice versus 2HP-β-CD with L-NAME-treated mice; *＃ p <* 0.05, PBS with L-NAME-treated mice versus 2HP-β-CD with L-NAME-treated mice. B, Immunohistochemical analysis of CD31 in ischemic hindlimbs of PBS-treated and 2HP-β-CD-treated mice with or without L-NAME. Representative images (upper panel) from calf muscle on postoperative day 28 are shown. Quantitative analysis of CD31-positive microvessel density is also shown (lower panel). Data represent the mean ± S.E.M. (n = 5 mice per group). * *p <* 0.05. Scale bar = 100 μm.

Systemic administration of 0.5 mg/ml L-NAME, a NOS inhibitor, to mice injected with PBS alone profoundly inhibited blood flow recovery in ischemic limbs (Fig 7A). Administration of L-NAME to mice injected with 2HP-β-CD abolished the increase in blood flow seen in these animals compared with those that had received only PBS. We confirmed that administration of the dose of L-NAME used in this study induced an increase in systolic blood pressure (119.9 ± 5.5 mmHg in control mice versus 142 ± 5.8 mmHg in L-NAME-treated mice), indicating the effectiveness of L-NAME. L-NAME administration inhibits 2HP-β-CD-induced increase in the microvascular density in ischemicmuscle as evaluated using anti-CD31 immunohistochemistry (Fig 7B).

## Discussion

In this study, we have investigated the functions and mechanisms of action of 2HP-β-CD in promoting angiogenesis both *in vitro* and *in vivo*. Our data indicate that 2HP-β-CD stimulated cell migration in both HUVECs and HASMCs. Treatment of cells with L-NAME significantly blocked migration of HUVECs, but not HASMCs. 2HP-β-CD stimulated proliferation of HUVECs and HASMCs, and the effect of 2HP-β-CD was inhibited by L-NAME in both cell lines (Fig 3). Local administration of 2HP-β-CD promoted blood flow and improved hindlimb muscle microcirculation in a mouse model of PAD induced by ligation of the femoral artery (Fig 4A). Further studies showed that local injections of 2HP-β-CD induced neovascularization in at least two ways: by angiogenesis and by recruitment of vascular smooth muscle cells (Fig 4B).

Angiogenesis, the process in which new vessels arise by branching from existing microvessels, involves the dissolution of the basement membrane berneath the endothelium together with endothelial cell (EC) migration and proliferation [32]. Several molecules, including VEGF [33]_,_ TGF-β [34,35], bFGF [34], hepatocyte growth factor (HGF) [36], angiopoietin (Ang-1) [37] and PDGF-B [38], have been shown to have angiogenic activity. Our data suggest that 2HP-β-CD stimulates angiogenesis through multiple mechanisms. Firstly, 2HP-β-CD is capable of acting on ECs to stimulate endothelial proliferation and migration both *in vitro* (Fig 3) and *in vivo* (Fig 4B). Secondly, 2HP-β-CD increased the expression of several angiogenic factors including VEGF-A and PDGF-BB, in HUVECs. VEGF-A, PDGF-B antibodies can block 2HP-β-CD-induced proliferation and migration of HUVECs (S1 Fig left panels).Injections of 2HP-β-CD also increased the expression of several angiogenic factors, including VEGF-A, PDGF-BB and TGF-β1, in ischemic muscle (Fig 4). VEGF-A is a specific EC mitogen, and it is also a heparin-binding glycoprotein [39], that can induce proliferation of ECs and *in vivo* angiogenesis [40]. TGF-β1 alone can induce EC survival and tubular network formation [41]. PDGF-BB modulates endothelial proliferation and angiogenesis *in vitro* via PDGF β-receptors [38].

bFGF can stimulate smooth muscle (SMC) proliferation [42], TGF-β1 can regulate SMC differentiation, PDGF is a potent chemoattractant for SMCs [43], and, during vessel formation, PDGF-BB is required for the recruitment and differentiation of smooth muscle-like cells [44]. In this study, 2HP-β-CD was shown to stimulate SMC proliferation and migration *in vitro* (Fig 3) by increasing the expression of bFGF (Fig 1), and the bFGF antibody can block 2HP-β-CD-induced proliferation and migration of HASMCs (S1 Fig right panels). Injections of 2HP-β-CD also increased the number of -smooth muscle actin (SMA)-positive blood vessels in ischemic muscle (Fig 4B, center). These results indicate that 2HP-β-CD promotes the formation of smooth muscle-covered blood vessels, i.e. arteriogenesis. Arteriogenesis generally involves growth and remodeling of preexisting collateral vessels or reflects *de novo* formation of mature vessels [45,31]. The maturation of blood vessels into multilayer structures is essential for their persistence.

These observations collectively indicate that administration of 2HP-β-CD stimulates neovascularization by promoting angiogenesis and vascular smooth muscle cell recruitment through both EC-autonomous and non-autonomous mechanisms.

It has been reported that VEGF-A can cause vasodilation through the NOS pathway in endothelial cells and activate cell migration [46]. It has also been demonstrated that VEGF enhances the expression of eNOS in native and cultured ECs, an effect that may be important in the process of VEGF-induced angiogenesis [47]. TGF-β1-mediated angiogenic activity has been shown to depend on phosphatidylinositol 3-kinase (PI3K)/AKT and p42/p44 mitogen-activated protein kinase (MAPK) signaling [41]. PDGF-B stimulation also produced an acute induction of TGF-β expression in SMCs through the MAPK/ERK pathway [43]. When human cultured airway SMCs were incubated with bFGF, there was an increase in ERK phosphorylation [48]. We have shown that 2HP-β-CD stimulates Akt and ERK both *in vitro* and *in vivo* (Fig 2 and Fig 5), 2HP-β-CD increased phosphorylation of Akt (Fig 2A) and eNOS (Fig 2B) in HUVECs, and the increase in phosphorylation of eNOS was effectively blocked by pharmacological inhibition with L-NAME (Fig 2B). Akt and ERK are key signaling molecules in cell proliferation, survival, migration and eNOS activation, and thereby angiogenesis. Injections of 2HP-β-CD also increased the extent of phosphorylation of eNOS at Ser^1177^, which is the Akt and ERK phosphorylation site critical for activation. Available evidence suggests that the eNOS/NO pathway contributes to angiogenesis [49–51]. The NOS inhibitor, L-NAME, significantly blocked 2HP-β-CD-induced HUVEC migration and proliferation (Fig 3). Administration of L-NAME reduced blood flow in both 2HP-β-CD-treated and PBS-treated (control) mice. 2HP-β-CD still stimulated blood flow in mice treated with L-NAME (Fig 7A), suggesting that mechanisms distinct from NO production could also participate in 2HP-β-CD-induced blood flow stimulation in ischemic limbs. L-NAME consistently inhibited 2HP-β-CD-induced angiogenesis (Fig 7B), suggesting that 2HP-β-CD-induced neovascularization is dependent on eNOS/NO. These observations also suggest that NO may increase blood flow by mechanisms other than stimulating neovascularization; NO-induced vasodilation may also be involved in stimulating blood flow in ischemic limbs [51]. The eNOS/NO-independent angiogenic actions of 2HP-β-CD likely involve Akt/ERK-mediated stimulation of EC proliferation and migration.

In conclusion, the data genarated in this study suggest that 2HP-β-CD significantly increases angiogenesis through mechanisms involving Akt/ERK-eNOS. Therapeutic angiogenesis with 2HP-β-CD may therefore be beneficial to patients with PAD.

## Supporting Information

S1 Materials (DOCX)

S1 Fig Effects of anti-VEGFA, PDGFB and bFGF antibodies on migration and proliferation of HUVECs and HASMCs induced by 2HP-β-CD. Figure A: HUVECs were treated with/without 10 ng/ml anti-VEGFA, PDGFB and bFGF antibodies, and HASMCs were treated with/without 1 ng/ml anti-VEGFA, PDGFB and bFGF antibodies followed by incubation with/without 10^–8^ M 2HP-β-CD for 72 h. Data represent the mean ± S.E.M. (n = 3 experiments). * p < 0.05, ** p < 0.01, vs. 10^–8^ M 2HP-β-CD group. Figure B: HUVECs were treated with/without 10 ng/ml anti-VEGFA antibody, 1 ng/ml anti-PDGFB antibody, and HASMCs were treated with/without 1 ng/ml anti-bFGF antibody followed by incubation with/without 10^–8^ M 2HP-β-CD for 5 h. Data represent the mean ± S.E.M. (n = 3 experiments). * p < 0.05, ** p < 0.01, vs. 10^–8^ M 2HP-β-CD group.
(TIF)
