# Supplementary material for: (2-Hydroxypropyl)-β-Cyclodextrin Is a New Angiogenic Molecule for Therapeutic Angiogenesis
Source: PLoS One. 2015 May 5;10(5):e0125323. doi: 10.1371/journal.pone.0125323 (PMC4420769; doi:10.1371/journal.pone.0125323)
Supplement: S1 Materials — (DOCX) [file pone.0125323.s001.docx]

**Supporting Information**

**S1 Materials**

**Cell Proliferation Test**

A cell proliferation assay was performed using Cell Counting Kit-8 (CCK-8) solution (Dojindo, Gaithersburg, MD) according to the manufacturer’s protocol. Briefly, HUVECs were seeded at a concentration of 500 cells/100 µl/well and HASMCs were seeded at a concentration of 1.0x10^3^ cells/100 µl/well in 96-well culture plates. The HUVECs were incubated with or without 10^-8^M 2HP-β-CD and 10 ng/ml anti-VEGFA antibody (Sigma-Aldrich, St. Louis, USA), 10 ng/ml anti-PDGFB antibody (Sigma-Aldrich) and anti-bFGF antibody (Sigma-Aldrich), and HASMCs incubated with or without 10^-8^M 2HP-β-CD and 1 ng/ml anti-VEGFA, PDGFB and bFGF antibodies for 72 h. The wells were treated with 10 µl/well of CCK-8 solution during the last 4 h of culture. Optical density (OD) of the wells was measured at 450 nm using a microplate reader (Thermo Scientific). This experiment was performed in triplicate.

**Transwell Migration Assay**

Transwell migration of HUVECs and HASMCs was determined using a 24-well transwell chamber with a pore size of 8 µm (Millipore, Massachusetts, USA). Cells (3x10^5^) in 200 μl of serum-free RPMI 1640 or DMEM containing 0.25% fatty acid-free BSA were loaded into the upper wells; the lower wells were filled with the same medium with or without 10^-8^M 2HP-β-CD and 10 ng/ml anti-VEGFA antibody and 1 ng/ml anti-PDGFB, anti-bFGF antibodies. The cells were allowed to migrate across the porous filter for 5 h at 37°C in a tissue culture incubator. After staining with 1% crystal violet and scraping the upper membrane surface with a cotton tip, the number of cells that migrated to the lower side of the filter was counted under a microscope in ten randomly selected high power fields. This experiment was performed in triplicate.
