# Supplementary figures and images for: (2-Hydroxypropyl)-β-Cyclodextrin Is a New Angiogenic Molecule for Therapeutic Angiogenesis
Source: PLoS One. 2015 May 5;10(5):e0125323. doi: 10.1371/journal.pone.0125323 (PMC4420769; doi:10.1371/journal.pone.0125323)

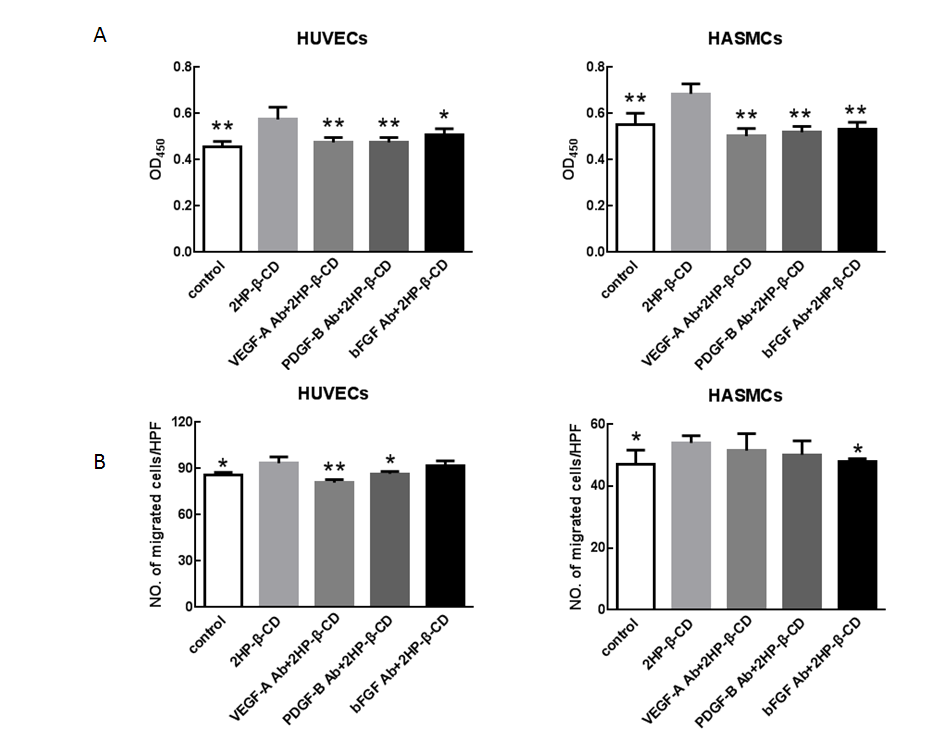

Supplement: S1 Fig — Figure A: HUVECs were treated with/without 10 ng/ml anti-VEGFA, PDGFB and bFGF antibodies, and HASMCs were treated with/without 1 ng/ml anti-VEGFA, PDGFB and bFGF antibodies followed by incubation with/without 10–8 M 2HP-β-CD for 72 h. Data represent the mean ± S.E.M. (n = 3 experiments). * p < 0.05, ** p < 0.01, vs. 10–8 M 2HP-β-CD group. Figure B: HUVECs were treated with/without 10 ng/ml anti-VEGFA antibody, 1 ng/ml anti-PDGFB antibody, and HASMCs were treated with/without 1 ng/ml anti-bFGF antibody followed by incubation with/without 10–8 M 2HP-β-CD for 5 h. Data represent the mean ± S.E.M. (n = 3 experiments). * p < 0.05, ** p < 0.01, vs. 10–8 M 2HP-β-CD group. (TIF) [file pone.0125323.s002.tif]
